# Novel naphthochalcone derivative accelerate dermal wound healing through induction of epithelial-mesenchymal transition of keratinocyte

**DOI:** 10.1186/s12929-015-0141-3

**Published:** 2015-07-01

**Authors:** Ga Young Seo, Manh Tin Ho, Ngoc Thuy Bui, Young Mee Kim, Dongsoo Koh, Youngho Lim, Changlim Hyun, Moonjae Cho

**Affiliations:** Department of Biochemistry School of Medicine, Jeju National University, Jeju, 690-756 South Korea; Department of Applied Chemistry, Dongduk Women’s University, Seoul, 136-714 South Korea; Division of Bioscience and Biotechnology, Konkuk University, Seoul, 143-701 South Korea; Department of Pathology, School of Medicine, Jeju National University, Jeju, 690-756 South Korea; Institute of Medical Science, Jeju National University, Jeju, 690-756 South Korea

**Keywords:** Naphthochalcone, Epithelial-mesenchymal transition, MMP, ECM

## Abstract

**Background:**

Wound healing is an intricate process whereby the skin repairs itself after injury. The epithelial-mesenchymal transition (EMT) is associated with wound healing and tissue regeneration. Naphthochalcone derivatives have various pharmaceutical properties. We investigated the effect of a novel naphthochalcone derivative, 2-(5-(2,4,6-trimethoxyphenyl)-4,5-dihydro-1H-pyrazol-3-yl)naphthalen-1-ol (TDPN), on dermal wound healing *in vivo* and the migration of keratinocytes *in vitro.*

**Result:**

We investigated the effect of TDPN on signaling pathway and epithelial-mesenchymal transition through protein and transcriptional expression. The TDPN treatment accelerated dermal closure about 3 days and remodeling of dermis. We found that treatment with TDPN induced the migration of keratinocytes but not cytotoxicity. TDPN induced the phosphorylation of ERK and AKT. TDPN-treated cells showed loss of adherence protein and showed induction of the transcriptional factor Slug, mesenchymal marker, and fibronectin. Moreover, TDPN treatment induced the expression of matrix metalloproteinase-1 (MMP-1), which degrades specific components of the extracellular matrix, thereby providing new substrates that facilitate migration and invasion. MMP expression is considered to be one of the major attributes acquired by cells after EMT.

**Conclusion:**

We propose that a novel naphthochalcone derivative TDPN is capable of promoting keratinocyte migration *via* the induction of EMT resulting acceleration of wound closure and matrix remodeling.

**Electronic supplementary material:**

The online version of this article (doi:10.1186/s12929-015-0141-3) contains supplementary material, which is available to authorized users.

## Background

Skin is the outermost organ of body that provides a barrier function to the body. In the epidermis layer of the skin, keratinocytes are the major cell population, constituting more than 90 % of all the cells [1]. When injuries to the skin occur, the keratinocytes at the wound site undergo morphologic alterations, changing from sedentary cells to migratory cells [2]. The phenotype of the migratory cells changes with respect to cell-cell adhesion and cell-matrix adhesion during the re-epithelialization stage. This phenotype partially resembles the epithelial to mesenchymal transition (EMT) process, which plays a critical role in cancer metastasis [2]. During both re-epithelialization and cancer metastasis, cells that undergo EMT lose contact with each other and the extracellular matrix (ECM) [3]. The ECM may be degraded by matrix metalloproteinases (MMPs), which represent a family of zinc-containing endopeptidases. The relationship between the EMT and MMPs has been clarified. MMP expression is considered to be one of the major attributes acquired by epithelial cells after they undergo the EMT [4]. MMPs, such as MMP-1, MMP-2, MMP-9, and MMP-13, are frequently associated with processes that involve tissue re-modeling and cell migration. Membrane type 1-MMP (MT1-MMP) is spatially and temporally regulated during MCF10A cell migration owing to its mediation of pericellular proteolysis of the laminin-5 (Ln-5) g2 chain [5]. The EMT is influenced by a wide range of regulatory factors. The signaling pathways for PI3K/AKT/mTOR and MAPK (ERK) are reported to be involved in the regulation of EMT [2, 6]. Increased phosphorylation of AKT has been implicated in the induction of the EMT and cell migration [7]. Furthermore, the transcriptional factors Snail and Slug play essential roles in cell migration. Slug expression has been shown to be up-regulated in the wound margins in the *in vitro*, *ex vivo*, and *in vivo* settings [8]. In Slug-null mice, re-epithelialization is reduced, compared with wild-type mice [8].

**Fig. 1:**
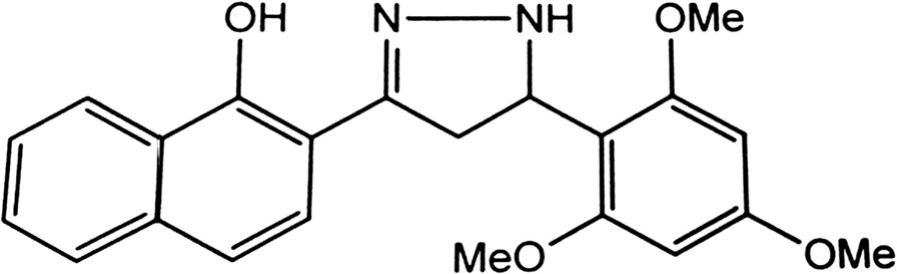
Structure of -(5-(2,4,6-trimethoxyphenyl)-4,5-dihydro-1H-pyrazol-3-yl)naphthalen-1-ol (TDPN)

Pure chalcones, which are extracted from plants, display interesting biological properties, such as antioxidant, cytotoxic, anticancer, and antimicrobial activities [9]. Naphthochalcone is a precursor to a variety of pharmaceutical agents. Naphthochalcone derivatives, which can be isolated from nature or biochemically synthesized, show various pharmaceutical effects [10]. One of the newly synthesized naphthochalcone derivatives, 1-(naphtho[2,1-*b*]furan-2-yl-carbonyl)-3,5-disubstituted-2,3-dihydro-1H-pyrazole, has been characterized by elemental analysis and in spectral studies. This compound has been evaluated for antimicrobial activity [11]. The compound (hydroxyphenyl) naphthol sulfonamide could be useful in optimizing 17β-HSD1 inhibitors for the treatment of endometriosis [12]. Another derivative, of methyl-1-hydroxy-2-naphthoate, may inhibit lipopolysaccharide-induced inflammatory responses in macrophages *via* suppression of the NF-κB, JNK, and p38 MAPK pathways [13]. Regarding structural analogues of the flavones, alpha-naphtoflavone promotes pro-collagen production in skin fibroblasts and is suggested to have anti-aging effects [14]. Recently we have screened series of compounds for promoting migration and proliferation of HaCaT cells and found that novel naphthochalcone derivative, 2-(5-(2,4,6-trimethoxyphenyl)-4,5-dihydro-1H-pyrazol-3-yl)naphthalen-1-ol (TDPN) (Fig. 1) had good activity.

The present study was aimed to examining whether novel naphthochalcone derivative (TDPN) exerts any effects on process of wound healing and, if so, what are the underlying mechanisms responsible for the action of TDPN. A murine excisional wound healing model *in vivo* and experiments using human keratinocyte cell line *in vitro* indicated TDPN accelerated wound closure by activating keratinocyte movement via inducing EMT like change. The results of this study provide novel drug candidate of cutaneous skin wound.

## Methods

### Reagent

The novel naphthochalcone derivative, 2-(5-(2,4,6-trimethoxyphenyl)-4,5-dihydro-1H-pyrazol-3-yl)naphthalen-1-ol (TDPN) (Fig. 1), was synthesized and kindly provided by Professor Youngho Lim (Division of Bioscience and Biotechnology, Konkuk University, Seoul, Korea). TDPN stock solutions were stored as aliquots at −20 °C, and were diluted to the final concentration before use.

### Cell culture

The human keratinocyte cell line HaCaT was cultured in RPMI medium that was supplemented with 10 % fetal bovine serum (FBS; GIBCO) and 1 % penicillin/streptomycin (PAA). Cells were incubated in a humidified atmosphere at 37 °C in 5 % CO_2_.

### Scratch wound healing assay

Because the doubling time of HaCaT cells is approximately 24 h, cells were seeded at 50 % confluency in culture dishes 24 h before the wound healing scratch assay. Therefore, the experiment was performed when cells reached monolayer formation. A scratch was made in the cell monolayer by drawing a sterile p-200 pipette tip across the surface of the culture dish. After the scratch was made, the culture medium was supplemented with TDPN. Dimethyl sulfoxide (DMSO; Amresco) treatment was used as the control. At 0 h and 24 h post-treatment, photographs of the cell monolayer were acquired at 4X magnification using the Olympus IX70 microscope equipped with a digital camera. The scratch was measured using the ImageJ software, and the difference between the initial and final width of the scratch was calculated.

### MTT assay

Cells were seeded on 96-well plates at 200 μl of cells which have density of 3 × 10^4^ cells/ml for each wells. Cells were treated with TDPN or DMSO for 24 h. MTT solution (10 μl of 5 mg/ml solution; Amresco) was added to each well and incubated for 37 °C for 4 h. Subsequently, the medium was gently removed and replaced with 150 μl of DMSO and incubated for 30 min with shaking to dissolve the precipitate. The samples were measured at an absorbance of 570 nm in a spectrophotometer [15].

### Western blotting

Cells were treated with TDPN or DMSO in time-dependent and dose-dependent manners. The cells were harvested by scraping and underwent lysis in RIPA buffer. The BCA method (Thermo Scientific) was used for protein concentration determination. The extracts were analyzed by SDS-PAGE followed by Western blotting with appropriate antibodies.

The following antibodies were used for Western blotting: p21 (catalogue no. 2947), AKT (9272), phosphorylated AKT (9271S), ERK (4695), Slug (9585S), and GAPDH (2118) were from Cell Signaling Technology; E-cadherin (610181) and Zo-1 (610966) were from BD Science Transduction; cyclin E (sc-247), cyclin D1 (sc-246), p53 (sc-126), phosphorylated ERK (sc-7383), collagen I (sc-25974), collagen III (sc-28888), and fibronectin (sc-9068) were from Santa Cruz Biotechnology; and MMP-1 (444209) was from Calbiochem. The secondary antibodies used in the Western blotting were anti-mouse (PI-2000; Vector Laboratories) anti-rabbit (PI-1000; Vector Laboratories), and anti-goat (AP-107P; Millipore). The blots were analyzed using the ImageJ software. The relative change in the ratio of the target protein to the DMSO control was determined.

### RT-PCR

Cells were seeded in a 60-mm culture dish and treated with TDPN. RNA from the treated cells was extracted using the Trizol reagent (Invitrogen). Then, 2 μg of total RNA were used to synthesize cDNA synthesis using a Reverse Transcriptase Kit (Promega). The resulting cDNA was used for RT-PCR using the G-Taq kit (Cosmo Genetech, Seoul, Korea) according to the manufacturer’s instructions.

RT-PCR was performed using the following gene-specific primers (forward and reverse primers, respectively), selected using the Blast Primer program: for *GAPDH*, 5′-GAAGGTGAAGGTCGGAGTC-3′ and 5′-GAAGATGGTGATGGATTTC-3′; for *p53*, 5′-ACACGCTTCCCTGGATTGG-3 and 5′-CTGGCATTCTGGGAGCTTCA-3′; for *p21*, 5′-GTCAGTTCCTTGAGCCG-3 and 5′-GAAGGTAGAGCTTGGGCAGG-3′; for *MMP-1*, 5′-AGGGGAGATCATCGGGAC-3′ and 5′-GGCTGGACAGGATTTTGG-3′; for *MMP-2*, 5′- AACACCTTCTATGGTGCCC-3′ and 5′-ACGAGCAAAGGCATCACCA-3′; and for *MMP-7*, 5′-TACAGTGGGAACAGGCTAGG-3′ and 5′-GGCACTCCACATCTGGGC-3′. The results were analyzed using the ImageJ software. The relative change in the ratio of the target protein to the DMSO control was determined.

### MMP zymography

MMP zymography was performed according to the method described by Gogly *et al*. [16], with the following modification: 8 % sodium dodecyl sulfate (SDS) gels that contained gelatin (0.01 mg/ml) were used. The SDS in the gels was removed by incubating the gels twice (30 min each) in 200 ml of 2.5 % Triton X-100 at 4 °C. Thereafter, the gel slabs were incubated at 37 °C overnight in the incubation buffer. The gels were then fixed and stained for 1 h with 0.05 % Coomassie Blue R-250. The molecular mass protein markers were readily visible as stained bands against the lighter blue color of the stained gelatin background. Gelatinase activity was apparent as clear zones of lysis (negative staining) against the blue background. The gels were scanned to create a permanent record of the results.

### Transwell invasion assay

The Transwell invasion assay was performed using a commercial Transwell plate (Corning). Cells were harvested by trypsinization and 7x10^4^ cells/well in 100ul of medium were seeded into the wells of the insert. The receiving wells were set up with medium containing FBS as a chemoattractant. After 24 h, media in insert wells was replaced by 100ul of free serum medium containing TDPN, TGF-β1, or DMSO. Invasive cells in the receiving wells were collected by trypsinization and counted by hemacytometer.

### Hoechst stain proliferation assay

The Hoeschst stain proliferation assay was performed as previously described [17]. 200 μl of cells at a density of 3 × 10^4^ cells/ml were seeded into the wells of 96-well plates. Cells were treated with TDPN or DMSO for 24 h. Hoechst 33342 solution (1 μl of 10 mM stock solution; Sigma) was added to each well and incubated for 37 °C for 30 min. Cell proliferation was estimated by direct measurement of changes in cell fluorescence using a spectrofluorometer (SPECTRAFLUOR, Tecan). The instrument was equipped with a 365-nm broadband filter for the excitation beam and a 450-nm narrowband interference filter plus a UV blocking filter for the emitted light

### ECIS migration assay

HaCaT cells were seeded at a density of 1 × 10^5^ cells/cm^2^ into ECIS arrays and impedance was measured using an ECIS instrument. Wounding pulses of 2400 mA were applied for 20 s at 6400 kHz. Media was then replaced by media containing TDPN or TGFβ1 or DMSO. The results were exported to the ECIS program.

### Trypan blue cell counting assay

5x10^4^ cells/well were seeded on 24-well plates. After 24 h of incubation, cells were treated with TDPN or DMSO in various concentrations for 24 h. All cells in each well were collected by trypsinization. The live cells were identified and counted by trypan blue staining. This experiment was repeated three times.

### Animal experiment

6-week old male ICR mice (n = 5 for each group: control and treatment TPDN) were chosen for the experiment. The fur was removed with an electronic hair clipper and removal cream. Dermal wound was placed on the middle of a back using an 8 mm punch instrument. In dermal wound site, we punch out whole dermis including subcutaneous fat layer (deep punch) and appendages of skin didn’t preserved. A few days later, granulation tissue and scab were formed at wound and we could confirm the epidermis and dermis were filled from surroundings of wound instead of granulation tissue. 200 μl of TDPN was applied to the wounds of the experimental group in concentrations of 200 μM (dissolved with Vaseline) for 20 days, while the control group was treated with same amount of DMSO (dissolved with Vaseline). Usually in vivo experiment, compounds have treated to animal approximately 100 ~ 200 times higher concentration of in vitro. Because process of the delivery and conversion of ingredients may cause lower of active material to reach to target points. However, in this study, the compound was applied to wound site directly, therefore we only applied just 10 times concentration of TDPN compared to in vitro. The wound was pictured every three day and the rate of wound closure was calculated as relative % of original wound area using Image J program. Skin samples were collected from mice at each of the wounding time points (9, 12, 15 days post-wounded) and fixed in 4 % formaldehyde. We cut formalin-fixed, paraffin-embedded tissues into 3um sections, stains all sections simultaneously by using routine hematoxylin–eosin (H-E) and Masson’s trichrome stain methods. The slides were reviewed by an Olympus BX51 microscope (Olympus Corp., Tokyo, Japan) with the apochromatic objective lens and a 0.85 numeric aperture.

## Results

### TDPN induces the migration but not the proliferation of keratinocytes

**Fig. 2:**
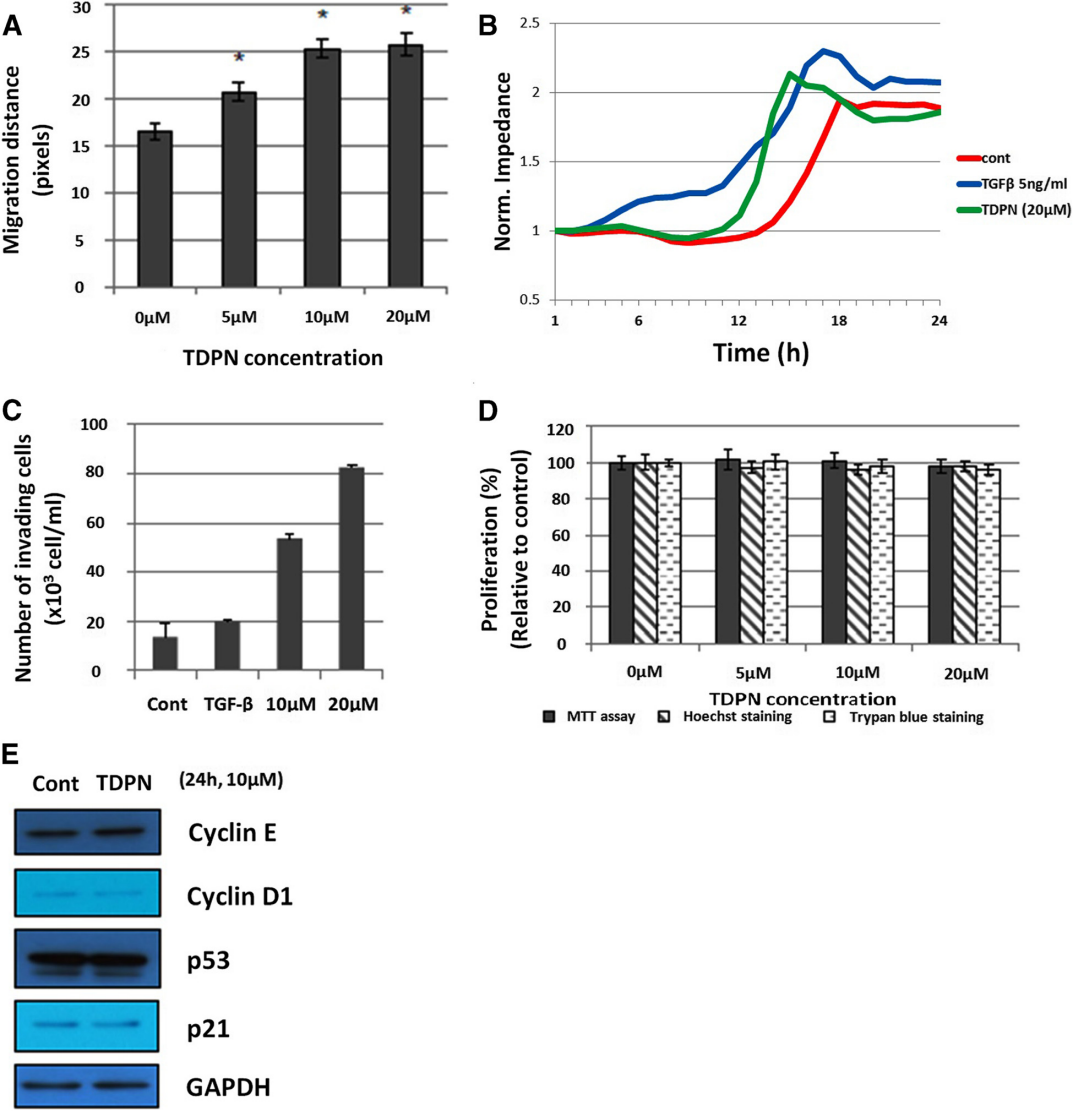
TDPN effects on migration and proliferation of HaCaT cell line. Migration of HaCaT cells were measured by scratch wound assay and ECIS system. Scratch wound healing assay of HaCaT cells treated with different concentrations of TDPN for 24 h. Cells were seeded with density 3 × 10^4^ cells/ml 24 h before scratch and treated. Distance of the scratch was measured after 24 h treated with TDPN (**a**). Cells were subjected to ECIS migration assay in the absence or the presence of TDPN or TGF-β as indicated. Cells were allowed to grow to confluence and initial resistance was measured for few hours. Cells were subjected to high voltage to initiate wound at 3 h resulting in the drop of resistance. Remaining cells were allowed to migrate in the presence or absence of TDPN and resistance changes were measured for 24 h after the creation of wound (**b**). Represented pictures are shown from 3 independent experiments. Invasion of TDPN treated cells were measured using transwell chambers as described in methods (**c**). Proliferation of keratinocyte cells were measures three different methods; MTT assay, Hoechst staining, and trypan blue counting assays (**d**). Cells were seeded for 24 h, then treated with various concentrations of TDPN or DMSO for 24 h. After treatment, cells were collected and counted in Trypan blue assay or incubated with Hoechst 33342 or MTT reagent. Results were measured by flourescence spectrometer or spectrometer. Western blot of cell cycle-related proteins comparing cells treated with 10 μM TDPN and untreated cells. Protein lysate from cells treated with TDPN for 24 h were taken using RIPA buffer. The expression level was measured by comparing the density of the target protein to GAPDH (**e**). (*)P < 0.05 compare to control group

Re-epithelialization involves the migration and proliferation of keratinocytes. In the present study, we investigated these two processes using the MTT assay and scratch wound healing assay for keratinocytes (HaCaT cells) treated with different concentrations of 2-(5-(2,4,6-trimethoxyphenyl)-4,5-dihydro-1H-pyrazol-3-yl)naphthalen-1-ol (TDPN) for 24 h. In the migration assay, TDNP promoted HaCaT cell migration dose-dependently up to 20 μM. However, there was little difference between 10 and 20 μM (Fig. 2a). It has been reported that TGF-β can induce EMT and migration of various cell types, including keratinocytes ([20, 31], Rasanen et al., 2010). To compare their abilities to induce migration, we performed migration assay comparing the ability of TGF and TDPN to induce migration, by both the ECIS system (Fig. 2b) and the Transwell invasion assay (Fig. 2c). These results demonstrated that TDPN induced migration and invasion comparably to TGF-β in the same time period of treatment.

Since migration rate may be influenced by proliferation rate, we tested cell growth. And keratinocyte proliferation was not affected by TDPN proven by three independent assays such as MTT assay, hemacytometer counting assay and in the proliferation Hoechst staining assay (Fig. 2d). These results also showed no significant changes between treated and untreated cells. To confirm the results, proteins related to the cell cycle were investigated in the treated cells. The levels of cyclin-dependent kinases E and D1, p53, and p21 were not significantly changed by TDPN (Fig. 2e). These data suggest that TDPN induces the migration but not the proliferation of keratinocytes.

### ERK and AKT phosphorylation is involved in promotion of keratinocyte migration by TDPN

**Fig. 3:**
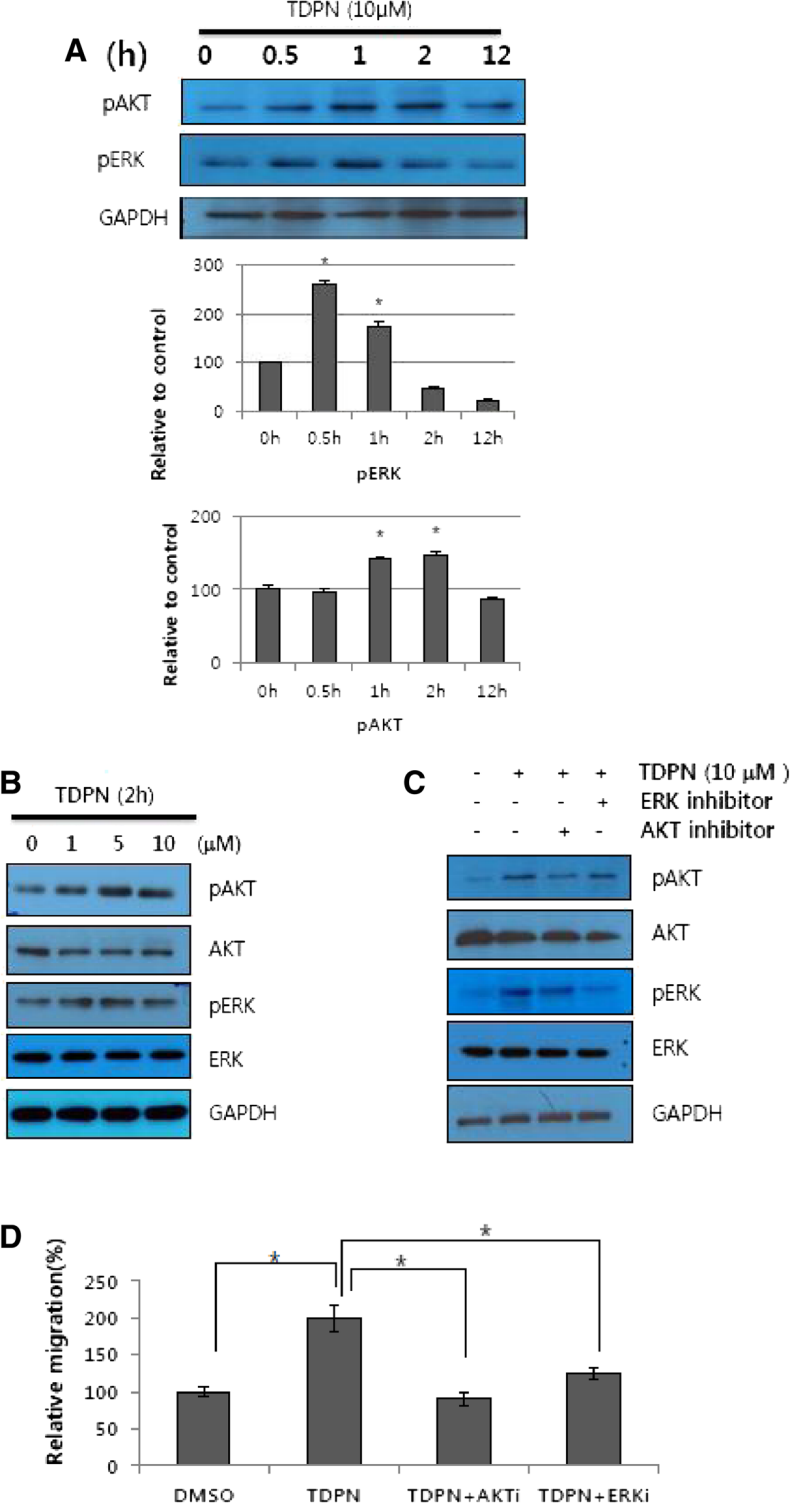
ERK and AKT phosphorylation is involved in promotion of keratinocyte migration by TDPN. HaCaT cells were treated with either DMSO or 10 μM TDPN for the indicated time (**a**) or the indicated dose for 2 h (**b**) and harvested for western blot analysis. Band intensity is presented as mean ± SE of relative to GAPDH and ratio between pAKT or pERK of TDPN and pAKT or pERK of control DMSO treatment from least 3 replications. (*)P < 0.05 compare to control group) (**c**) Cells were pre-treated with PI3K inhibitor (LY49002, 50 μM,) or MEK inhibitor (pd98059, 50 μM,) for 24 h, then treated with TDPN for 1 h and harvested for western blot analysis, to assess the involvement of the ERK and AKT signaling pathways. (**d**) After co-treatment of TDPN and PI3K inhibitor (LY49002, 50 μM) or MEK inhibitor (pd98059, 50 μM) for 24 h, scratch wound healing assay were performed. Distance of the scratch was measured relative to control after 24 h of treatment with TDPN. (*)P < 0.05)

It is well-known that mitogen-activated protein (MAP) kinase family members, ERKs, as well as the PI3K/AKT pathway, are important for the regulation of cell migration. Therefore, we investigated the expression levels of regulatory proteins related to cell migration, such as AKT and ERK. Keratinocytes were treated with TDPN for different time periods and protein lysates were assayed for phosphorylation of AKT and ERK. We used a stock of TDPN containing approximately 0.05 % DMSO. Each time point, cells treated either DMSO or TDPN in DMSO were collected and assayed by western blot analysis. The relative intensity between TDPN and control group at each specific time point was calculated (Fig. 3a). Within 2 h ERK and AKT signals were activated. These signals were increased dose-dependently at 2 h time point (Fig. 3b) and the treatment of specific inhibitors against ERK and AKT attenuate TDPN induced phosphorylation (Fig. 3c). To identify the TDPN induced migration of keratinocyte was mediated by ERK and AKT signaling, we performed a wound healing assay in which cells were pretreated with a PI3K inhibitor (LY49002, 50 μM) and a MEK inhibitor (PD98059, 50 μM) then treated with TDPN or left untreated. The results demonstrated that inhibiting the AKT and ERK pathways abolished the effects of TDPN on migration (Fig. 3d).

### TDPN induces EMT-like change

**Fig. 4:**
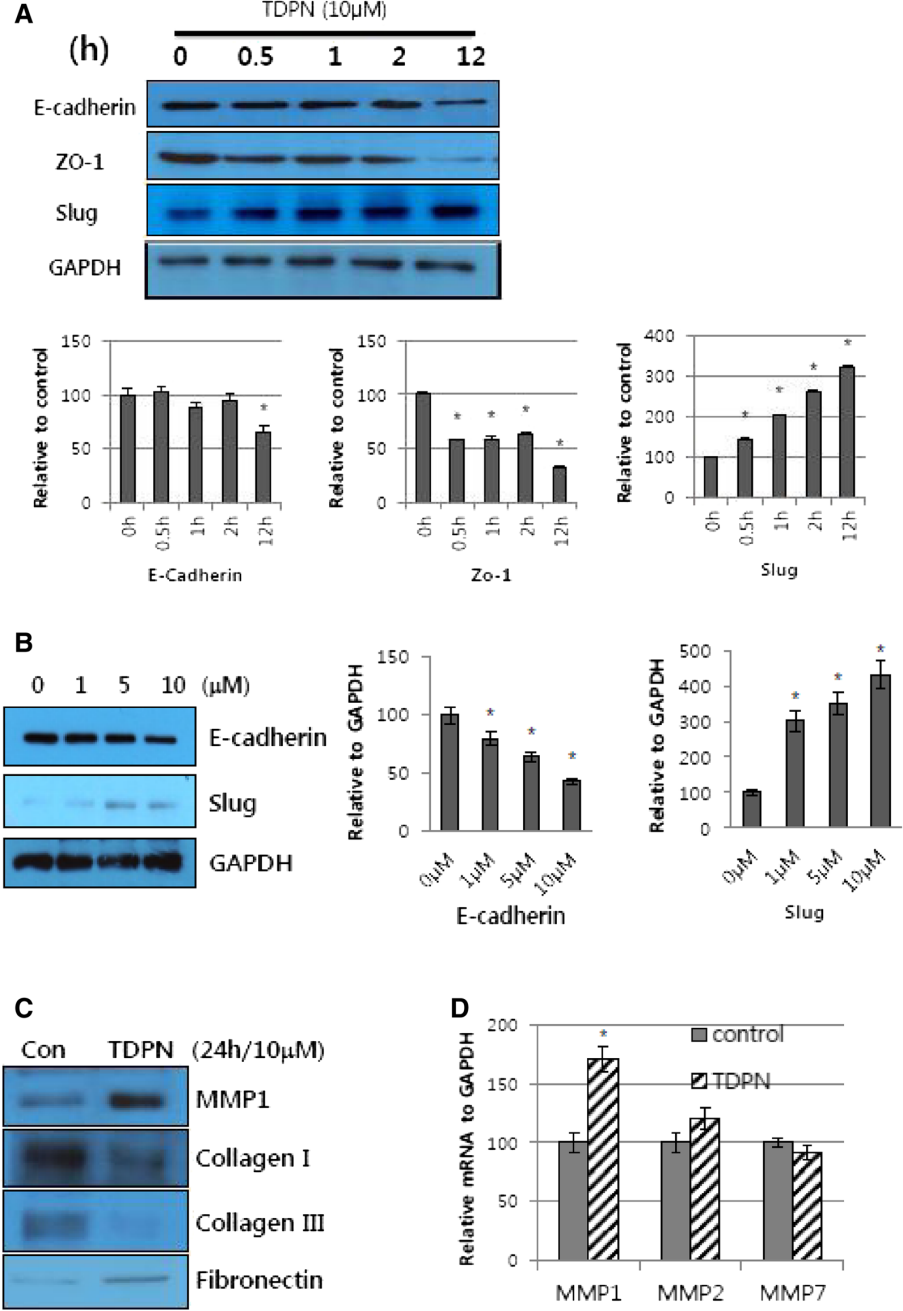
TDPN induces EMT like change. HaCaT cells were treated with TDPN (10 μM) for the indicated time (**a**) or at the indicated dose (**b**) and harvested for western blot analysis. (data presented as mean of relative to GAPDH ± SE from least 3 replications, (*)P < 0.05 compare to control group). (**c**) HaCaT cells treated with 10 μM TDPN for 24 h. After treatment, the conditioned media from the cells was concentrated using amicon centrifugation. Protein in media was collected and analysed by western blot for Collagen I, Collagen III, Fibronectin, and MMP1. (**d**)Transcriptional expression of MMPs in HaCaT cells treated with TDPN (10 μM for 24 h). After treatment, total RNA from each cells was taken by Trizol reagent and analysed by RT-PCR for MMP1, MMP2, MMP7. GAPDH was used as a control. (data presented as mean of relative to GAPDH ± SE from least 3 replications, (*)P < 0.05 compare to control group)

It has been reported that EMT occurs in keratinocytes at wound sites during the re-epithelialization stage [18, 19]. Thus, we investigated whether TDPN promotes re-epithelialization through EMT. We treated TGF-β1 (10 ng/ml) and TDPN for 24 h and observed morphological and biochemical changes. On above condition, morphological changes were not dramatic (Additional file 1: Figure S1). It has been reported that TGF-β induces invasion and changes the cell size of several cell types including epithelial cells and keratinocytes [20-22]. However, morphological change of keratinocytes by TGF-β on *in vitro* varies depending on cell types. HaCaT cells treated with TGF-β1 for 24 h only form actin stress fibers but morphology does not change [23]. However, in cellular level, main EMT markers such as epithelial marker proteins Zo-1 and E-cadherin, and mesenchymal marker, transcriptional factor Slug changed (Fig. 4a). Western blot results revealed that TDPN treatment increased Slug expression and decreased the E-cadherin and Zo-1 in a time-dependent manner (Fig. 4a) and dose-dependent manner (Fig. 4b). This suggests that TDPN treatment causes the loss of cell-cell adhesion. In addition, the level of the mesenchymal marker fibronectin was slightly increased (Fig. 4d).

In both EMT tumors and migrating keratinocytes, degradation and remodeling of the ECM are needed. The EMT pathway has been reported to involve MMP expression [4]. In the present study, zymography revealed that the levels of MMP-2 and MMP-9 were not changed by TDPN treatment (Additional file 2: Figure S2). However, the Western blot results for TDPN-treated cells showed that the MMP-1 protein level was significantly increased (Fig. 4c). The transcriptional expression pattern showed a similar result (Fig. 4d).

### TDPN accelerated dermal wound healing

**Fig. 5:**
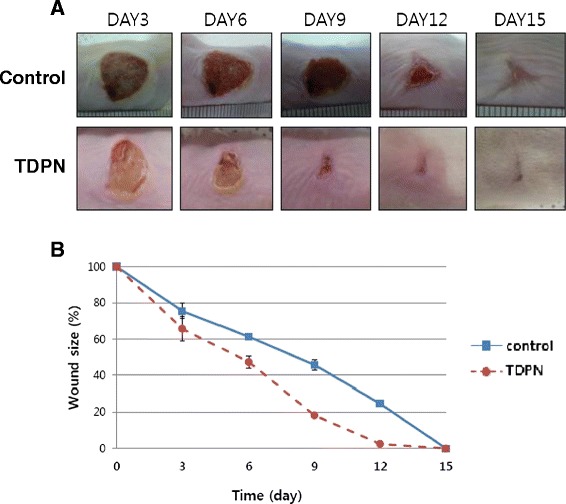
Wound closure after a full-thickness dermal excision. Male ICR mice (6 weeks of age) underwent a full-thickness 8 mm excisional wounding. They were either muck treated or treated with cutaneous application of TDPN (200 μM) daily. The wound closure was pictured every three day (**a**). The averages of opened wound area were measured by image program and plotted as relative % of original wound (**b**). The values were expressed as means ± SE from 6 wounds in each group till day 9, 4 wounds at day12 and 2 wounds at day 15

**Fig. 6:**
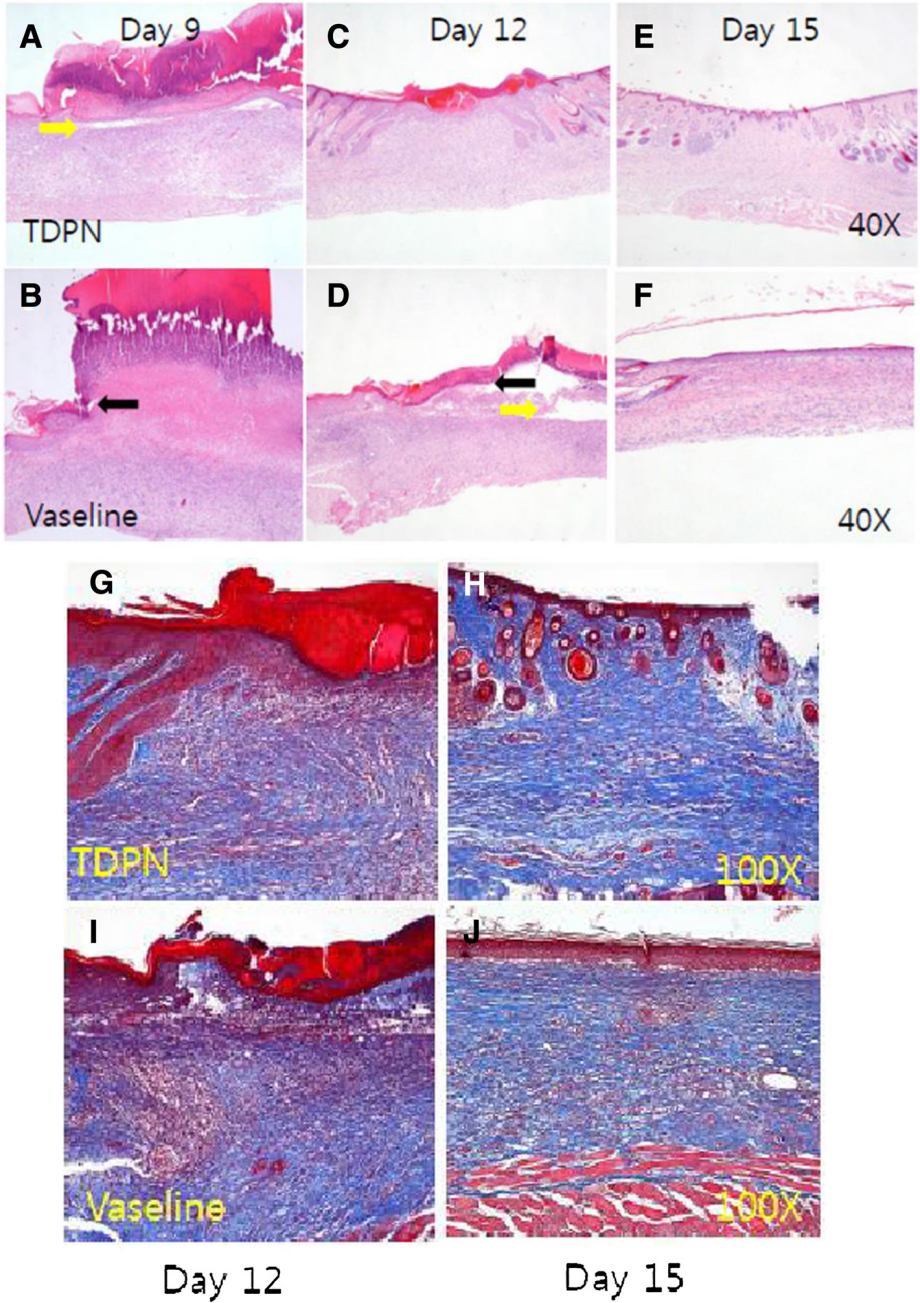
Histological findings of dermal wound tissues during the healing process. Dermal specimens were obtained from ICR mice either TDPN treated (**a**, **c** and **e**) or muck treated (**b**, **d**, and **f**) as in Fig. 5, at day 9 (**a** and **b**), day 12 (**c** and **d**) or day 15 (**e** and **f**). They were subjected to H-E staining (**a**-**f**) or Masson Trichrome staining (**g**-**j**). Yellow arrow indicated open area. Black arrow indicated epidermal leading edge. Represented pictures are shown from 2 wounds in each group at each day

Full-thickness excisional wound were made on the dorsal of mice and TDPN or Vaseline were applied to wound site topically every day. Hereby, we investigated the effects of TDPN on skin remodeling phase. It could affect scar formation and faster resolving collagen deposit. TDPN treated mice exhibited a faster wound closure then muck treated animals as early as day 9 (Fig. 5a). The average of opened wound area were measured by image program and the rate of closure of TDPN treated was 3 day faster than untreated ones (Fig. 5b). Tissue samples were taken at day 9, 12 and 15 post-wounded and performed H&E staining. On day 9, TDPN treated wound revealed that epidermal closure is complete but dermal closure is not sufficient to have open area (yellow arrow) and inflammation and necrosis is still remain in outer skin (Fig. 6a). Whereas muck treated wound showed that necrosis and inflammation accompanied in wound site and epidermal leading edge is observed (black arrow) but epidermal closure is not complete. The granulation tissue formation in dermis is incomplete and dermal closure is ongoing (Fig. 6b). On day 12, in TDPN treated wound, epidermal and dermal closure is complete and hair follicle start to show but not yet start differentiation. Contraction of granulation tissue squeezes hair follicle to wound direction (Fig. 6c). In muck treated wound, granulation tissue formation is processed and dermal closure is complete, however, the proliferation of epidermis is not sufficient and the epidermal closure is incomplete (Fig. 6d). On day 15, in TDPN wound, epidermal and dermal closure is complete perfectly and glands differentiation is processing throughout the wound site (Fig. 6e). Day 15 muck treated wound still have no sign of a nodule that resembled early sweat or sebaceous glands (Fig. 6f). Collagen deposits were stained by Masson Trichrome (Fig. 6g-j). In TDPN treated animal, until day 12 collagen deposition is still ongoing and distribution of collagen fiber is uneven (Fig. 6g) however on day 15, collagen density is high and evenly distributed as normal dermis (Fig. 6h). In muck treated wound, the collagen deposit did not show much difference compared to TDPN treated (Fig. 6i-j). Results suggested accelerated wound closure may attributed by faster epidermis differentiation.

### TDPN promote epidermis development via keratinocyte activation

**Fig. 7:**
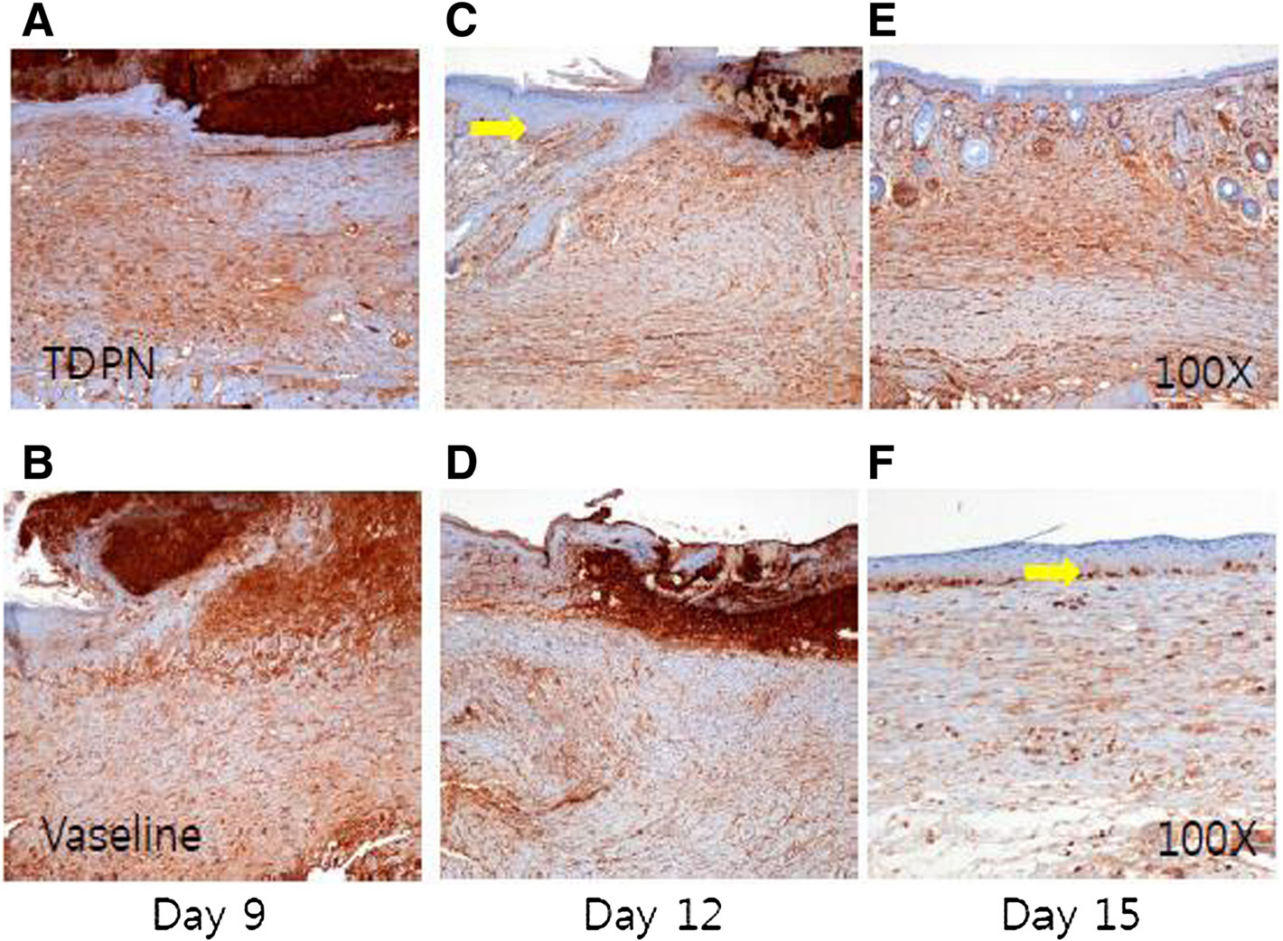
Proliferation of dermal wound tissues during the healing process. Dermal specimens were obtained from ICR mice either TDPN treated (**a, c** and **e**) or muck treated (**b**, **d**, and **f**) as in Fig. 5, at day 9 (**a** and **b**), day 12 (**c** and **d**) or day 15 (**e** and **f**). Proliferation of cell in wound site was measured by ki-67 antibody staining. Represented pictures are shown from 2 wounds in each group at each day

We performed Ki-67 antibody staining for detect proliferation (Fig. 7). In both case, the day 9 show highest proliferation index (Fig. 7a-f). In the TDPN group at day 12 and muck treated group at day 15, cells located at the bilayer of epidermis (yellow arrow) show strong reaction as seen in normal epidermis (Fig. 7b,f). However, on day 15, TDPN treated group show high proliferation not only epidermal bilayer but also around nodules that resembled early sweat or sebaceous glands (Fig. 7c). These results suggested that TDPN mainly activated epidermal keratinocyte rather than dermal fibroblast.

## Discussion

Our results reveal that the novel naphthochalcone derivative TDPN show faster wound closure and matrix remodeling (Figs. 5 and 6) via promoting the migration but not the proliferation of keratinocytes (Fig. 2). Importantly, we observed promotion of the EMT pathway in the TDPN-treated cells. The expression levels of the adherence junction protein E-cadherin and tight junction protein ZO-1 were reduced in the cells treated with TDPN. Also, the expression levels of the transcriptional factor Slug, as well as those of the mesenchymal marker fibronectin were induced in these cells (Fig. 4). Furthermore, the ERK and AKT signaling pathways were found to be involved in TDPN induction of EMT, as evidenced by the induction of phosphorylation of ERK and AKT which was confirmed by pretreatment AKT inhibitor (LY49002, 50 μM) or ERK inhibitor (PD98059, 50 μM) attenuated effect of TDPN (Fig. 3). As a consequence of the EMT, MMP-1 (but not MMP-2 or MMP-9) was found to be significantly induced. Following the induction of MMP-1 in the TDPN-treated cells, the ECM proteins collagen I and collagen III were significantly degraded.

The TDPN mainly stimulate keratinocyte not fibroblast. Keratinocyte migration, a part of cell proliferation stage, plays an important role during the wound healing [24]. Several approaches have been used to study new compounds and new pathways that promote migration and wound healing [25, 26]. For example, Protection effects of green tea extract (EGCG) against UV were found to be involved in the proliferation of normal human keratinocytes [27]. EGCG-induced Erk phosphorylation and activation of the Akt pathway was found to promote keratinocyte survival [28]. However, in our study, TDPN showed effects on the migration but not on the proliferation of keratinocytes. VPA cutaneous wound healing by increasing the motility of HaCaT keratinocytes through ERK and phosphatidylinositol 3-kinase (PI3-kinase)/Akt signaling pathways [28].

The epithelial-mesenchymal transition (EMT) is one of the major factors that affect cell migration associated with wound healing and tissue regeneration [18, 19]. Loss of cell junctions is considered to be a crucial marker for EMT [17]. Extensive previous studies have focused on how tight junctions are down-regulated in EMT [29, 30, 5]. In addition, during the early stage of re-epithelialization, when cells undergo EMT, reduced cell-cell contacts allow the cells to migrate. EMT down-regulation of the cell junction protein E-cadherin appears to involve regulation by Wnt or TGF-β *via* inducible activation of kinase pathways that modulate GTPase, Smads, PI3Ks, MAP kinases, β-catenin, and activate transcription factors, including LEF-1, Snail, Slug, and Scatter, ultimately leading to repression of the *E-cadherin* gene [31]. Apart from the loss of cell junctions, the increased expression levels of Slug and mesenchymal marker act as a marker for EMT [19, 32]. Slug, a key transcription factor is responsible for the down-regulation of E-cadherin in both explanted human skin and primary keratinocytes [33].

The cell migration is regulated by various signaling pathways. The MAPK signaling pathways are associated with cell migration [34], as well as the EMT pathway [35, 36], and the PI3K/AKT signaling pathway has also been implicated in cell migration [6, 37] and EMT [38]. TDPN induced phosphorylation of ERK and AKT in time- and dose-dependent manners (Fig. 3). A previous report showed that transforming acidic coiled-coil protein 3 (TACC3) induced EMT and migration through induction of the AKT and ERK pathways [37]. Furthermore, it has been reported that inhibition of the ERK and AKT pathways leads to inhibition of the induction of EMT by TGF-β1 [39]. Our results revealed that the phosphorylation of signaling pathway proteins AKT and ERK were induced early in TDPN treatment, while changes of EMT markers such as Slug E-cadherin detected later. We suggest that TDPN triggers early signals in the EMT pathway (Figs. 3 and 4).

Matrix metalloproteinases (MMPs), which are extracellular proteases that are highly expressed at wound sites, degrade specific components of the ECM, thereby providing new substrates that facilitate migration and invasion [40]. The relationships between EMT and MMPs have been described. MMP expression is considered to be one of the major attributes acquired by epithelial cells after EMT [4]. MMP-2 and MMP-9 have been reported to play essential roles in the EMT in the avian embryo [41, 42]. Interestingly, our enzymatic activity results do not accord with the results in the previous report, in that the levels of MMP-2 and MMP-9 were not changed in our treated cells. However, MMP-1 (type I collagenase) is needed in the epidermis for re-epithelialization [40]. In human oral keratinocytes, MMP-1 mRNA was expressed while the keratinocytes covered the wound surface [43]. In agreement with those results, MMP-1 was induced in the TDPN-treated cells in the our study (Fig. 4). *In vitro* keratinocyte culture, collagen I and collagen III were significantly degraded in the TDPN treatment group. Given that MMP-1 is a collagenase, its induction would lead to the degradation of the collagen helping keratinocyte migration at the margin of dermis.

## Conclusion

The finding of novel compound – TDPN – to promote wound healing via keratinocyte migration supplies a good tool for studying wound healing mechanism and possible drug candidate.

## Additional files

Additional file 1: Figure S1. Morphology of HaCaT cell change when treated with TDPN for 24 h. TGF-β1 (5 μM for 24 h) was used as a positive control. Additional file 2: Figure S2. Gelatin zymograph assay using media from HaCaT cells treated with 10 μM TDPN for 24 h. After treatment, the conditioned media from the cells was concentrated using amicon centrifugation.

## Abbreviations

TDPN: 2-(5-(2,4,6-trimethoxyphenyl)-4,5-dihydro-1H-pyrazol-3-yl)naphthalen-1-ol
EMT: Epithelial-mesenchymal transition
TGF-β1: Transforming growth factor beta 1
ECM: Extracellular matrix
MMP: Matrix metalloproteinases
