# Supplementary figures and images for: Novel naphthochalcone derivative accelerate dermal wound healing through induction of epithelial-mesenchymal transition of keratinocyte
Source: J Biomed Sci. 2015 Jul 1;22(1):47. doi: 10.1186/s12929-015-0141-3 (PMC4488135; doi:10.1186/s12929-015-0141-3)

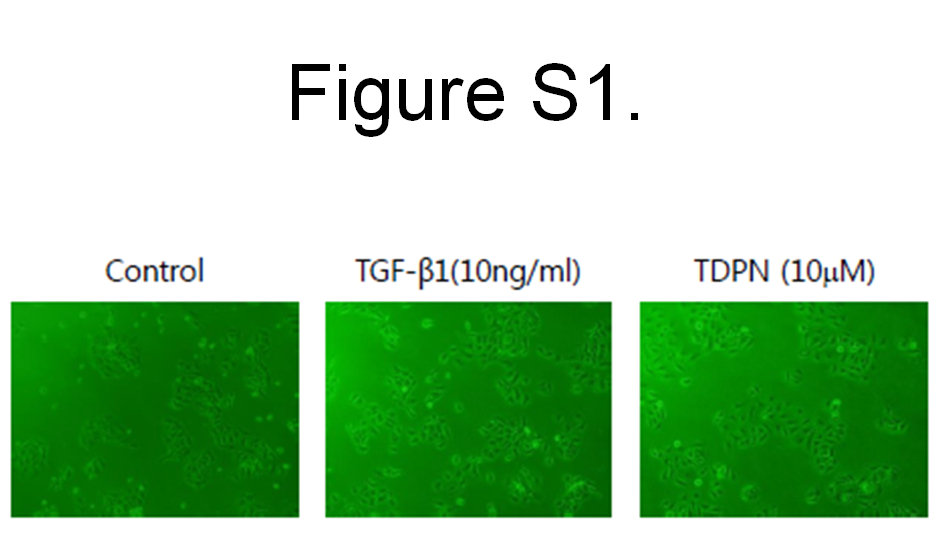

Supplement: Additional file 1: Figure S1. — Morphology of HaCaT cell change when treated with TDPN for 24 h. TGF-β1 (5 μM for 24 h) was used as a positive control. [file 12929_2015_141_MOESM1_ESM.doc]

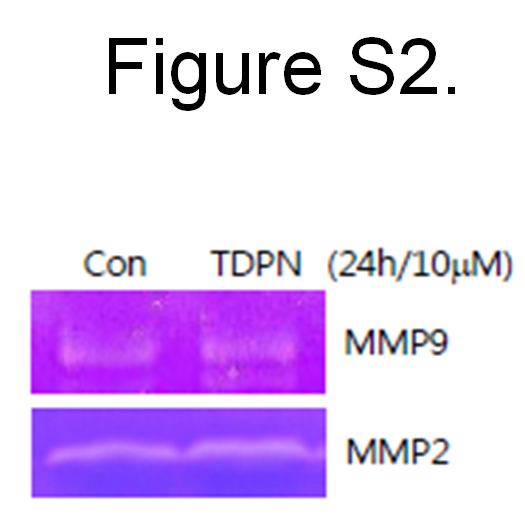

Supplement: Additional file 2: Figure S2. — Gelatin zymograph assay using media from HaCaT cells treated with 10 μM TDPN for 24 h. After treatment, the conditioned media from the cells was concentrated using amicon centrifugation. [file 12929_2015_141_MOESM2_ESM.doc]
